# The Oligomeric State of Rad6-Rad18 and Its Interactions with Translesion Synthesis Proteins

**DOI:** 10.3390/biom16081172

**Published:** 2026-08-12

**Authors:** Tyler J. Woodward, Brittany M. Ripley, Justin A. Ling, M. Todd Washington

**Affiliations:** Department of Biochemistry, University of Iowa, Iowa City, IA 52242-1109, USA; tjwoodward@uiowa.edu (T.J.W.); brittany_ripley-dajles@bio-rad.com (B.M.R.); jling@kumc.edu (J.A.L.)

**Keywords:** DNA replication, DNA damage, DNA repair, mutagenesis

## Abstract

Translesion synthesis is a major DNA damage bypass pathway in which specialized DNA polymerases, such as pol η and Rev1, are recruited to stalled replication forks where they catalyze nucleotide incorporation opposite DNA damage. Translesion synthesis is regulated by the Rad6-Rad18 complex, which catalyzes PCNA mono-ubiquitylation. Despite its central role in regulating translesion synthesis, its oligomeric state and molecular interactions are poorly understood. Here, we use mass photometry to show that Rad18 co-purifies with Rad6 and forms a series of larger oligomeric complexes. Using yeast two-hybrid studies, we showed that while the full-length Rad18 complex interacts with pol η, its interaction with Rev1 is controlled by auto-inhibition. The release of this auto-inhibition is associated with the dissociation of the Rad6-Rad18 complex, freeing Rad18 monomers and dimers. Because pol η participates in non-mutagenic translesion synthesis, while Rev1 participates in mutagenic translesion synthesis, this auto-inhibition likely allows pol η preferential access to stalled replication forks by delaying Rev1 recruitment. Such an ordered polymerase-recruitment mechanism would reduce the likelihood of mutations.

## 1. Introduction

DNA damage is problematic because it causes DNA replication forks to stall, which can lead to genomic instability, cell death, and carcinogenesis [1,2,3,4,5,6]. To avoid these challenges, eukaryotes possess several DNA damage bypass pathways. One major bypass pathway, translesion synthesis (TLS), utilizes specialized DNA polymerases [7,8,9]. These polymerases, such as DNA polymerase (pol) η and Rev1, are recruited to stalled replication forks and catalyze nucleotide incorporation opposite DNA damage [10,11,12,13]. TLS is regulated by the mono-ubiquitylation of proliferating cell nuclear antigen (PCNA), an essential replication accessory factor that functions as a sliding clamp during DNA replication [14,15]. Mono-ubiquitylation is catalyzed by an enzyme complex comprising the E2 ubiquitin conjugating enzyme, Rad6, and the E3 ubiquitin ligase, Rad18 [14,15]. Despite the key role that the Rad6-Rad18 complex plays during DNA damage bypass, the oligomeric state and interaction with TLS polymerases are poorly understood.

**Figure 1:**
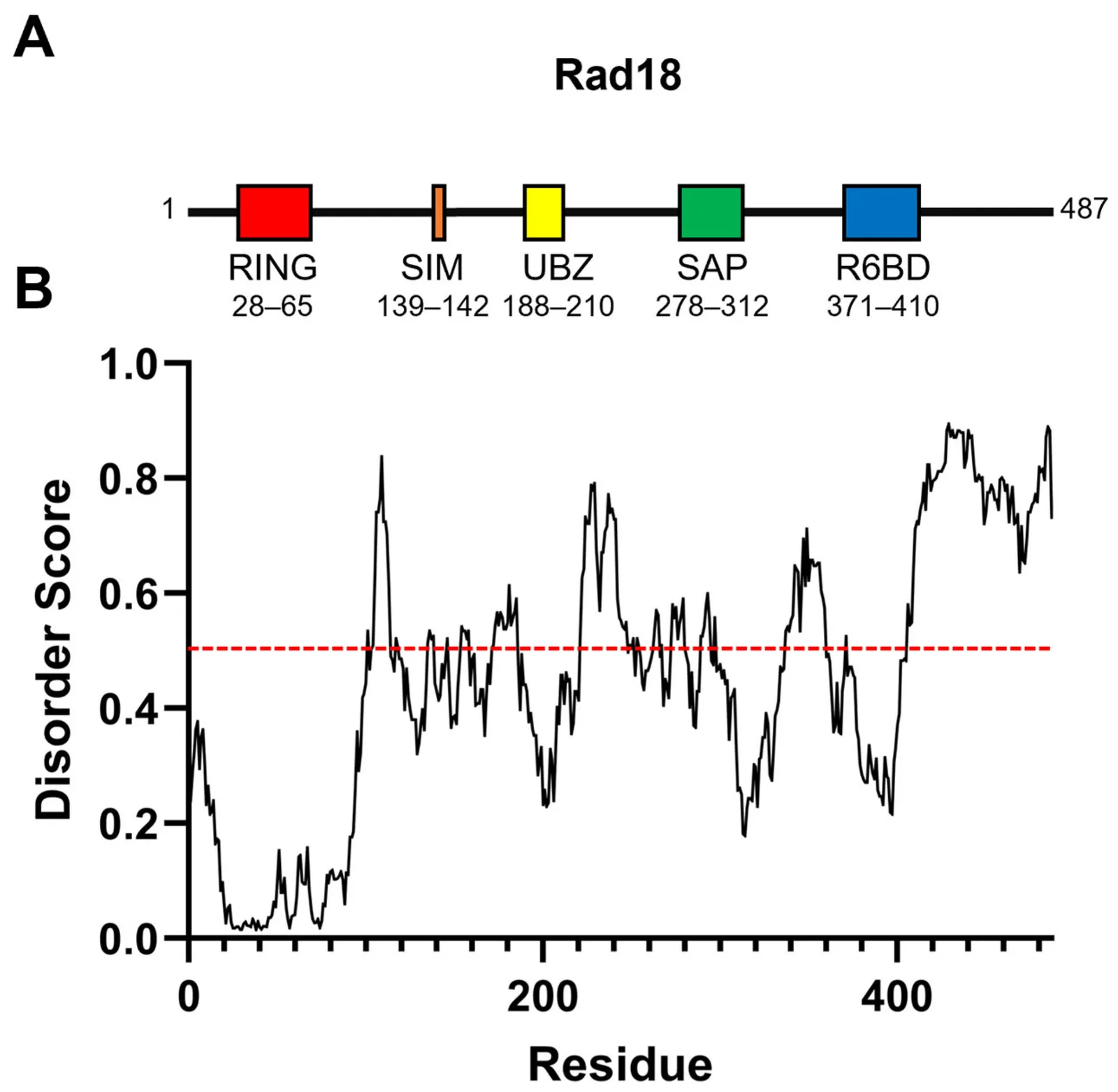
Rad18 Domain schematic and structural prediction. (**A**) Domain schematic of Rad18 showing discrete domains interspersed with intrinsically disordered regions. (**B**) Disorder plot of Rad18 generated with IUPred2A [24,25]. The red line represents the 0.5 disordered score.

The structure of Rad6 has been determined via X-ray crystallography, revealing it is a globular protein with a characteristic E2 fold [16,17]. By contrast, the structure of Rad18 is not well understood. The structures of three small regions within Rad18 have been determined via NMR and X-ray crystallography. These regions are the RING domain; the ubiquitin-binding, zinc-binding (UBZ) domain; and the Rad6-binding domain (R6BD) [18,19,20,21]. In addition to these small regions, Rad18 possesses a SAF-A/B, Acinus, and PIAS (SAP) domain and a SUMO-interacting motif (SIM). SAP domains are well-characterized by their DNA-binding function [22]. The presence of the SIM suggests that Rad6-Rad18 may be a SUMO-directed ubiquitin ligase [23]. The remaining regions of Rad18, however, remain structurally uncharacterized. Disorder prediction tools suggest that these regions are predominantly disordered (Figure 1) [24,25].

Our previous studies have provided compelling evidence that in addition to catalyzing the mono-ubiquitylation of PCNA, the yeast Rad6-Rad18 complex is also directly involved in regulating TLS polymerase recruitment. For example, the yeast Rad6-Rad18 complex interacts with pol η, which may facilitate the recruitment of this TLS polymerase to stalled replication forks [26]. To better understand how Rad6-Rad18 may recruit TLS polymerases, we examined the interactions between Rad18 and translesion synthesis polymerases pol η and Rev1. Using yeast two-hybrid assays, we found that full-length Rad18 interacts with pol η, but not Rev1. By contrast, we showed that removal of the Rad18 R6BD or a specific amino acid substitution in Rad18 (W387A) restores interaction with Rev1, while eliminating its interaction with pol η. We hypothesized that these mutations likely correlate with a disruption of the Rad6-Rad18 oligomeric state. Using mass photometry, we found that wild-type Rad18 co-purifies with Rad6 and forms a series of larger undetermined oligomeric complexes. Furthermore, we showed that the Rad18 W387A mutant protein fails to co-purify with Rad6 and exists predominantly as free monomers. Taken together, the results support a model in which the oligomeric state of the Rad6-Rad18 complex regulates the sequential recruitment of TLS polymerases. Specifically, the formation of larger, oligomeric complexes auto-inhibits its interactions with Rev1 and favors interactions with pol η. Given that Rev1 is more mutagenic than pol η [7,8,9], the auto-inhibition of the interaction of Rad6-Rad18 and Rev1 may delay the recruitment of Rev1 to stalled replication forks, thereby reducing the likelihood of mutations.

## 2. Materials and Methods

### 2.1. Yeast Two-Hybrid

The genes encoding yeast pol η (*RAD30*), Rev1, RPA (*RFA1*), and Rad18 were ligated into yeast two-hybrid fusion vectors pGBKT7 and pGADT7 containing the Gal4 transcriptional activator domain or the Gal4 DNA-binding domain, respectively [27,28]. All truncation or mutant constructs were made using the Q5 mutagenesis kit (New England Biolabs). All of the plasmids used are listed in Supplemental Table S1. The yeast two-hybrid plasmids were transformed into AH109 cells and plated on media lacking leucine and tryptophan (-LW). Colonies were grown overnight in omission media (-LW) at 30 °C and plated on omission media (-LW) at dilutions of 10^8^, 10^7^, and 10^6^ cells/mL. To test for interaction between the two proteins, the yeast cells were also plated on omission media lacking histidine, leucine, and tryptophan (-HLW) in dilutions of 10^8^, 10^7^, and 10^6^ cells/mL. Growth on histidine-deficient media indicates interactions between the tested protein constructs.

### 2.2. Expression and Purification for Rad6-Rad18 and Rad6

Yeast *RAD6* was cloned into YIp128 (*LEU2* selectable marker) under an ADH1 promoter. Yeast *RAD18* was cloned into pYES2 (*URA3* selectable marker) and was translationally fused to an N-terminal 6xHis tag under a GAL1 promoter. These plasmids (pKW831 and pKW832, respectively) were transformed together into BJ5464 cells, and the proteins were overexpressed by growing cells at 30 °C overnight in omission media (-Leu, Ura) without glucose. For the expression and purification of Rad6-Rad18, solid galactose was added to a final concentration of 2% when cells reached saturation in the absence of glucose and were grown for 6.5 h. After expression, cells were pelleted via centrifugation at 6000 rpm for 20 min at 4 °C.

Cell pellets were resuspended in a lysis buffer (50 mM Tris, pH 7.6, 150 mM NaCl, 10% glycerol). Cells were lysed using a C3-Avestin Emulsiflex homogenizer (Ottawa, ON, Canada) in the presence of DNase, PMSF, and a Roche Complete Protease Inhibitor Cocktail. The Rad6-Rad18 complex was purified using a series of affinity chromatography and size-exclusion chromatography steps. All buffers used contain 150 mM HEPES, pH 7.6, 150 mM sodium chloride (unless specified otherwise), 2 μM ZnSO_4_, and 10 mM beta-mercaptoethanol. The cell lysate was cleared by centrifugation at 16,000 rpm for 1 h at 4 °C. The cleared lysate was loaded onto a 5 mL His-Trap HP Ni Sepharose HP column (Cytiva, Marlborough, MA, USA) and eluted using a linear gradient of 10 mM to 400 mM imidazole. Next, fractions were combined and loaded onto a 5 mL Hi-Trap Heparin Sepharose HP column (Cytiva). Proteins were eluted using a linear gradient of 100 mM to 1.5 M sodium chloride. Finally, fractions were combined and loaded onto a Hi-Load Superdex S200 size-exclusion chromatography column (Cytiva). Size-exclusion utilized an isocratic buffer containing 25 mM arginine, 25 mM glutamic acid, and 10% glycerol. The purity of the protein was verified using SDS-PAGE. Afterwards, the protein was flash frozen in liquid N_2_ and stored at −80 °C. The Rad18 W387A mutant was generated via a Q5 mutagenesis kit (New England Biolabs, Ipswich, MA, USA). The newly generated plasmid (pKW847) and Rad6-expressing plasmid (pKW831) were co-transformed into BJ5464 yeast cells and subjected to the same expression and purification as wild-type Rad18.

For expression and purification of Rad6 alone, pKW831 was transformed into BJ5464 cells and plated on omission media plates (-Leu). Cells were grown in omission media at 30 °C until mid-phase was reached. Afterwards, cells were pelleted via centrifugation at 6000 rpm for 20 min at 4 °C. Cell pellets were resuspended in a lysis buffer (50 mM Tris, pH 7.5, 150 mM NaCl, 10 mM beta-mercaptoethanol, 10% glycerol). Cells were lysed using a C3-Avestin Emulsiflex homogenizer in the presence of DNase, PMSF, and a Roche Complete Protease Inhibitor Cocktail. The protein was purified using anion-exchange and size-exclusion chromatography. All buffers used contain 50 mM Tris, pH 7.5, 150 mM sodium chloride (unless specified otherwise), 10 mM beta-mercaptoethanol, and 10% glycerol. The cell lysate was cleared by centrifugation at 16,000 rpm for 1 h at 4 °C. The cleared lysate was loaded onto a 10 mL Q-Sepharose HP column (Cytiva) and eluted using a linear gradient of 100 mM to 1 M sodium chloride. Finally, fractions were combined and loaded onto a Hi-Load Superdex S200 size-exclusion chromatography column (Cytiva). Size-exclusion utilized an isocratic buffer. The purity of the protein was verified using SDS-PAGE. Afterwards, the protein was flash frozen in liquid N_2_ and stored at −80 °C.

### 2.3. Mass Photometry

Mass photometry experiments were performed using a Refeyn Two Mass Photometer (Refeyn Ltd., Oxford, UK) as described previously [29]. Briefly, microscope coverslips (High Precision Deckgläser No. 1.5H) and silicon gaskets (Grace Bio-Labs, Bend, OR, USA) were cleaned by serial rinsing with ultrapure water and HPLC-grade isopropanol (Sigma-Aldrich, St. Louis, MO, USA) followed by drying with a filtered air stream. Mass photometry measurements were performed at room temperature using Dulbecco’s phosphate-buffered saline without calcium and magnesium (Thermo Fisher, Waltham, MA, USA). Movies were acquired for 60 s using AcquireMP (version 2.3.0) and were processed and analyzed using DiscoverMP (version 2.3.0). A β-Amylase and thyroglobulin protein standard was used for mass calibration. After applying the calibration, mass counts were tabulated into a frequency distribution. Three replicate measurements were then averaged to produce the final frequency distribution graphs.

### 2.4. Biolayer Interferometry

Biolayer interferometry (BLI) experiments were performed using an Octet RED96 instrument from Sartorius (Göttingen, Germany). Biotinylated peptides for the isolated wild-type and W387A R6BD were purchased from GenScript (Singapore). Wild-type and W387A R6BD peptides corresponding to residues 384–408 of Rad18 were synthesized with an N-terminal biotin modification. The wild-type peptide sequence was Biotin-RKGWMVMFRKDFARLIREAKMKIKT, while the W387A peptide sequence was Biotin-RKGAMVMFRKDFARLIREAKMKIKT. Lyophilized peptides were resuspended in BLI-100 buffer (20 mM Tris, pH 7.5, 100 mM NaCl, 1 mM EDTA, and 0.05% Tween-20). Streptavidin biosensors were soaked in BLI-100 buffer for 30 min prior to data collection. Data collection utilized a 5-step protocol: equilibration (180 s in BLI-100 buffer), loading (60 s with 10 μg/mL of biotinylated peptide), baseline (60 s in BLI-100), association (300 s in purified Rad6 proteins at various concentrations), and dissociation (180 s in BLI-100 buffer). Double-reference subtraction was performed by including control experiments with unloaded streptavidin biosensors and peptide-loaded biosensors incubated in buffer lacking Rad6. Sensorgrams were processed and analyzed using Data Analysis software (version 11.1), and figures were generated using GraphPad Prism (version 11).

### 2.5. Genetic Complementation Studies

The wild-type *RAD18* gene under control of its native promoter was cloned into yeast shuttle vector pTB364 (*LEU2* selectable marker) to generate pKW833. Site-directed mutagenesis was performed on the resulting plasmid to generate a gene encoding the W387A Rad18 mutant protein, pKW834. Plasmids were transformed into yeast strain BY4742 (*rad18*∆), and the resulting strains were grown on synthetic media lacking leucine at 30 °C. Overnight cultures in omission media were diluted to 10^7^, 10^6^, 10^5^, and 10^4^ cells/mL and plated on omission media. These plates were exposed to 0, 25, 50, and 100 J/m^2^ of ultraviolet (UV) radiation and grown in the dark at 30 °C for 3 days. Site-directed mutagenesis was conducted on plasmid pKW833 to create a truncated form of Rad18 (aa 1–363). The R6BD (wild-type and W387A mutant) genes were cloned into pESC-Ura under a galactose-inducible promoter, Gal1. Double plasmid transformations were conducted in yeast strain BY4742 (*rad18*∆). The resulting strains were grown on synthetic media lacking leucine and uracil at 30 °C. Overnight cultures in omission media (lacking leucine, uracil, and glucose; containing 2% galactose) were diluted to 10^7^, 10^6^, 10^5^ and 10^4^ cells/mL and plated on omission media (with glucose). These plates were exposed to 0, 25, 50, and 100 J/m^2^ of ultraviolet (UV) radiation and grown in the dark at 30 °C for 4 days. All experiments were performed in biological triplicate. Semi-quantitative survival scoring was performed independently by two blinded evaluators. One point was assigned for each serial dilution exhibiting visible growth, and an additional 0.5 point was awarded when only partial growth was observed at the highest dilution showing detectable growth. Scores from both evaluators were averaged and tabulated to generate a final survival score for each sample/condition (Supplemental Figures S1–S5).

## 3. Results

### 3.1. Interactions of the Rad6-Rad18 Complex with Translesion Synthesis Proteins

**Figure 2:**
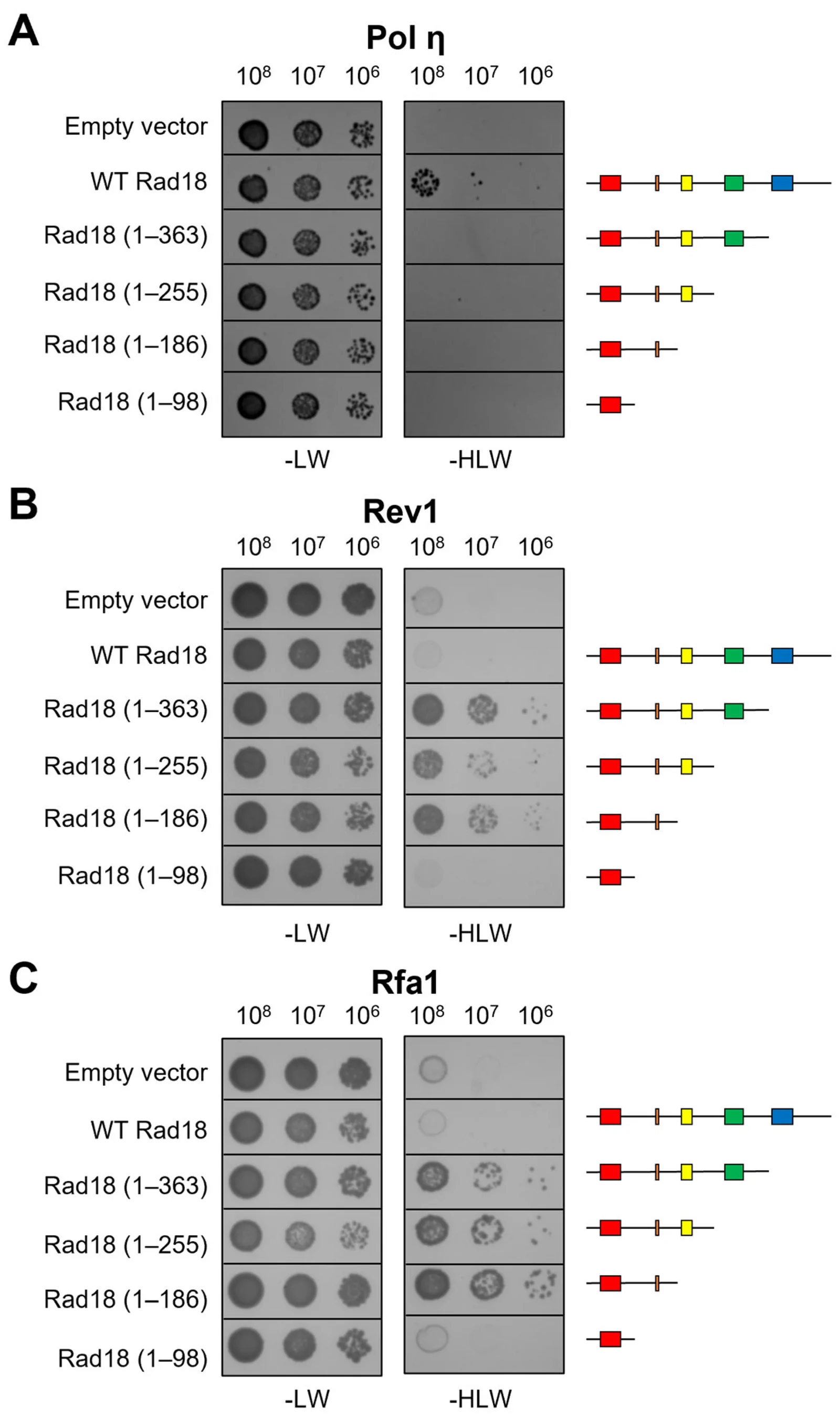
Yeast two-hybrid assay of Rad18 interaction with pol η, Rev1, and Rfa1. (**A**) Omission media plates with serial dilution of AH109 cells expressing the yeast two-hybrid system, testing for interaction between pol η and Rad18 (full-length and a series of nested C-terminal truncations), with controls. Growth on -HLW indicates interaction between pol η and full-length Rad18, either directly or indirectly. (**B**) Omission media plates testing for interaction between Rev1 and Rad18 (full-length and a series of nested C-terminal truncations), with controls. Growth on -HLW indicates interaction between Rev1 and truncated Rad18, suggesting potential auto-inhibition. (**C**) Omission media plates testing for interaction between Rfa1 and Rad18 (full-length and a series of nested C-terminal truncations), with controls. Growth on -HLW indicates interaction between Rad18 and Rfa1, in a comparable manner to Rev1.

We previously demonstrated that the yeast Rad6-Rad18 complex interacts with the translesion synthesis DNA polymerase (pol) η [26]. Additionally, truncated forms of Rad18 have been demonstrated to interact with the single-stranded binding protein replication protein A (RPA) through the Rfa1 subunit [30,31]. To further examine the interactions between the yeast Rad6-Rad18 complex and translesion synthesis polymerases, including Rev1, we carried out yeast two-hybrid studies. In a Gal4-based yeast two-hybrid assay, growth on histidine-deficient media indicates an interaction between the tested protein constructs, either through direct or indirect interactions. Consistent with previous reports, we found that full-length Rad18 interacts with pol η (*Rad30*; Figure 2A). By contrast, full-length Rad18 did not interact with Rev1 (Figure 2B). We hypothesized that Rev1 may interact with truncated forms of Rad18, similar to previous reports with Rfa1. As a result, we replicated the results with Rfa1 and found that Rfa1 interacts only when the R6BD of Rad18 was removed (Figure 2C). Interestingly, this same phenomenon was observed when we examined the ability of truncated forms of Rad18 to interact with Rev1. Thus, while full-length Rad18 does not interact tightly with either Rev1 or Rfa1, the removal of the R6BD from the C-terminus of Rad18 allows for interactions with these proteins. This suggests that both Rev1 and Rfa1 interact with Rad18 between residues 98 and 363, which contains the SIM, the UBZ domain, and the SAP domain. This also suggests that the presence of the R6BD of Rad18 acts to auto-inhibit the interactions of Rad18 with Rev1 and Rfa1. However, we found that pol η loses its interaction with truncated forms of Rad18, suggesting the R6BD is required for the engagement between pol η and Rad18.

**Figure 3:**
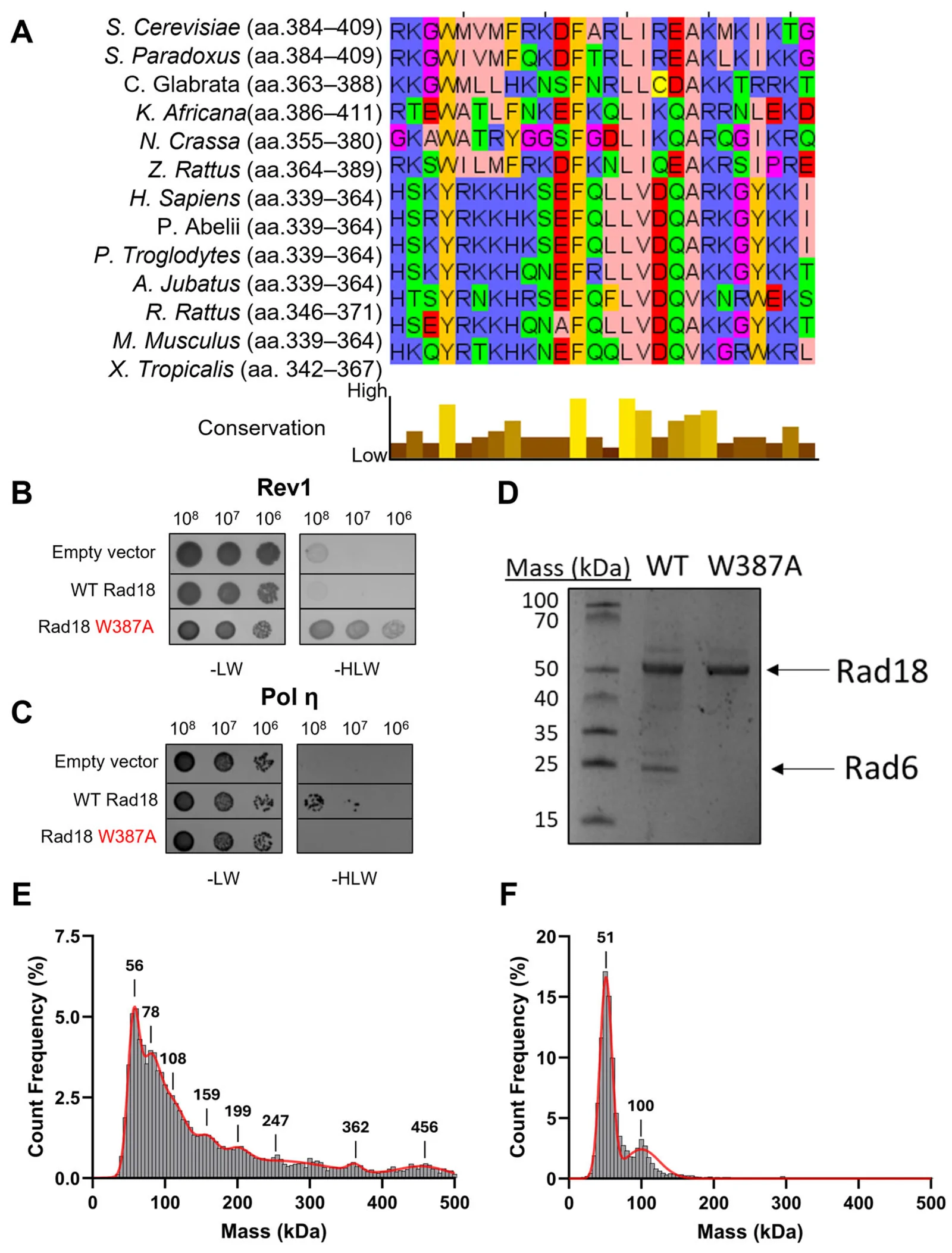
Identification, characterization, and expression of the Rad18 W387A mutant. (**A**) Protein sequence of the Rad6-Binding Domain (R6BD) in Rad18 across several eukaryotic species. Several positions are well conserved across species. (**B**) Omission media plates with serial dilution of AH109 cells expressing the yeast two-hybrid system, testing for interaction between Rev1 and wild-type Rad18 or Rad18 W387A, with controls. Growth on -HLW indicates interaction between Rev1 and Rad18 W387A. (**C**) Omission media plates with serial dilution of AH109 cells expressing the yeast two-hybrid system, testing for interaction between pol η and wild-type Rad18 or Rad18 W387A, with controls. Growth on -HLW indicates interaction between pol η and wild-type Rad18. (**D**) Polyacrylamide gel showing the co-purification of the Rad6-Rad18 complex and the Rad18 W387A mutant. (**E**) Purified wild-type Rad6-Rad18 complex (20 nM) was subjected to mass photometry in three replicates. The frequency of counts for each replicate was averaged. A majority of the complex corresponds to Gaussian curves centered at 56 and 78 kDa, which are similar to the expected monomeric Rad18 molecular weight (~56 kDa) and a one-to-one complex of Rad6-Rad18 (~76 kDa). Other Gaussian distributions were identified centering at 108, 159, 199, 247, 362, and 456 kDa. (**F**) Purified Rad18 W387A (20 nM) was subjected to mass photometry. A majority of the counts correspond to a Gaussian distribution centering at 51 kDa, similar to the expected monomeric mass of Rad18 (~56 kDa). In addition, a Gaussian near the expected dimeric molecular weight (~112 kDa) was identified at 100 kDa, representing a minority of the total counts.

Because the R6BD of Rad18 acts to block its interactions with Rev1 and Rfa1, we first examined the conservation of the R6BD across several eukaryotic species (Figure 3A). We identified several positions that appear to be highly conserved across species, suggesting potential importance in inter- or intra-molecular interactions. Then, we created a series of amino acid substitutions in the R6BD to determine if we could identify a single amino acid substitution that would release this inhibition to the same extent as the removal of the R6BD (Supplemental Figure S6). Using yeast two-hybrid assays, we identified a single amino acid substitution of the conserved aromatic residue, tryptophan-387 to alanine (W387A), that released inhibition of the interactions of Rad18 with Rev1 (Figure 3B). Interestingly, this single amino acid substitution ablates the interaction between Rad18 and pol η (Figure 3C). Overall, the W387A mutant appears to release inhibition with Rev1 and disrupt the interaction with pol η, which is akin to the removal of the R6BD.

### 3.2. Composition of the Rad6-Rad18 Protein Complex

To further examine the effects generated by the W387A mutation in Rad18, we expressed both wild-type Rad18 and the W387A mutant Rad18 in the presence of overexpressed Rad6. Using an affinity tag on Rad18, we then purified Rad18 and assessed for co-purification of Rad6 to determine if these proteins had a stable interaction. For wild-type Rad18, we could detect both Rad6 and Rad18 on the polyacrylamide gel (Figure 3D). We then used mass photometry to directly examine the oligomeric state of the purified wild-type Rad6-Rad18 complex in solution (Figure 3E). From the histograms recorded one minute after dilution and loading on the mass photometer, we observed several peaks. After averaging histograms of counts across three replicates and fitting the data to a sum of Gaussians, the most abundant calculated mean values for these peaks are 56 and 78 kDa. These peaks corresponded to the expected masses of a monomeric Rad18 (56 kDa) and a one-to-one Rad6-Rad18 complex (77 kDa). In addition, several punitive higher order species were observed. We suggest these represent various oligomeric states of the Rad6-Rad18 complex, but we are unable to definitively identify the stoichiometries of these species given the overlapping Gaussian tails. Incidentally, Rad6 alone, which is 22 kDa, is too small to be detectable by mass photometry. Overall, these results demonstrate that the Rad6-Rad18 complex co-purifies as a complex and exists in various oligomeric states.

For the W387A mutant of Rad18, we detected only Rad18 on the polyacrylamide gel despite being co-expressed with Rad6 (Figure 3D). We then analyzed the oligomeric state of the Rad18 W387A mutant protein using mass photometry (Figure 3F). After averaging histograms of counts across three replicates and fitting the data to a sum of Gaussians, we observed two peaks, one major and one minor, with mean values of approximately 51 kDa (the major peak) and 100 kDa (the minor peak). These peaks corresponded to the expected masses of a single Rad18 protein alone (56 kDa) and a small amount of a dimeric complex containing two Rad18 subunits (112 kDa). Overall, the W387A mutant of Rad18 fails to co-purify with Rad6 and fails to form higher-order oligomeric species, at least to the same extent as wild-type Rad18. Taken together, this suggests that the W387A substitution releases auto-inhibition by destabilizing the Rad6-Rad18 complex, forming predominantly free Rad18 monomers and homodimers.

### 3.3. Mechanism of Auto-Inhibition by the R6BD

**Figure 4:**
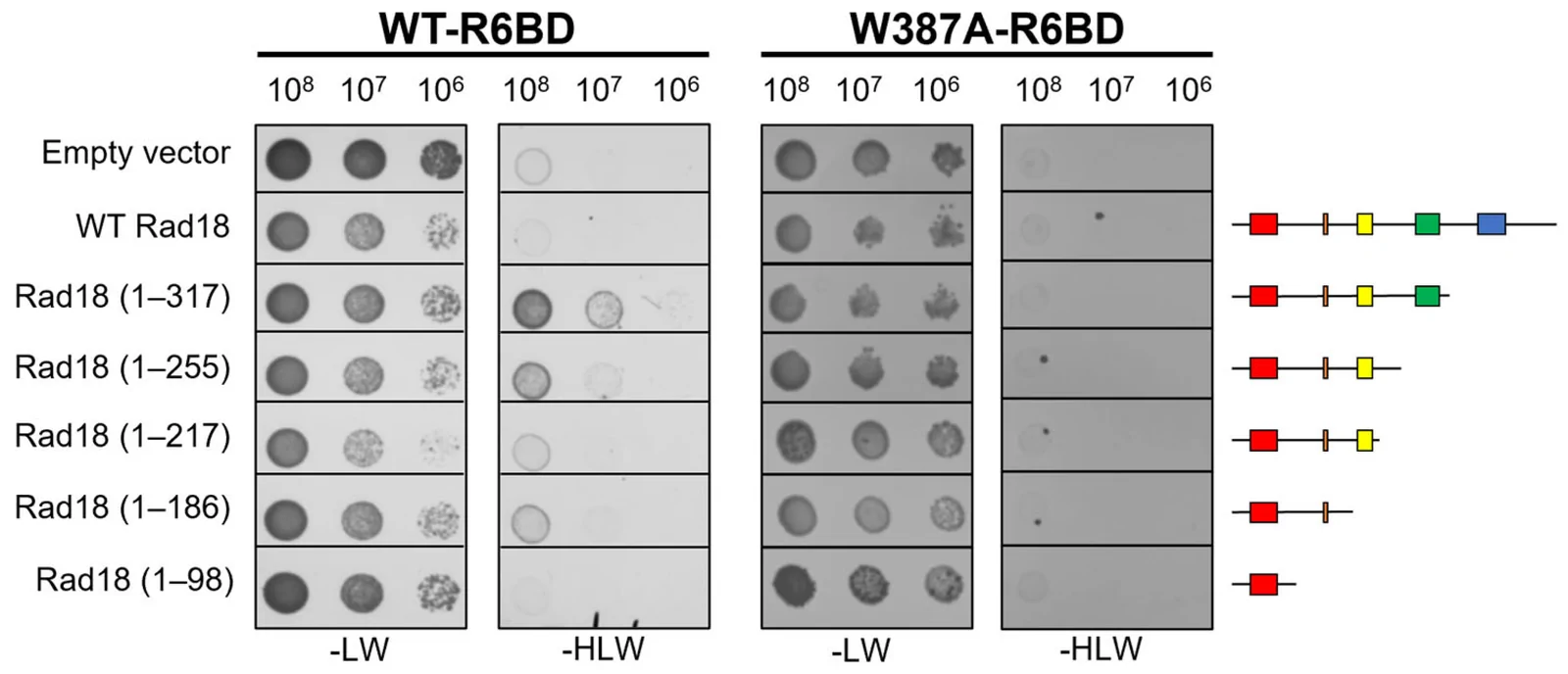
Yeast two-hybrid assay of Rad18 or truncated form interaction with Rad6-Binding Domain (R6BD) in trans. Omission media plates with serial dilution of AH109 cells expressing the yeast two-hybrid system, testing for interaction between Rad18 (wild-type and truncated forms) and Rad6-binding domain (R6BD), including wild-type and W387A mutation, with controls. Growth on -HLW indicates interaction between truncated forms of Rad18 and the wild-type R6BD, suggesting auto-inhibition is caused by an intramolecular interaction within the Rad6-Rad18 complex.

We next tested the idea that the R6BD acts as an auto-inhibitory domain that makes an intramolecular interaction with another region of the Rad6-Rad18 complex. Previous studies have demonstrated that the R6BD binds to Rad6 [21]. Additionally, there is evidence that the RING domain also interacts with Rad6 [32]. We hypothesize that both of these regions (RING and R6BD) are required to facilitate Rad6-Rad18 complex formation. If this is the case, we would expect the R6BD to complement truncated Rad18 constructs in trans by engaging another region of the Rad6-Rad18 complex, thereby promoting complex formation. To test this claim, we used yeast two-hybrid experiments to test for interaction between Rad18 and the R6BD in trans. Interestingly, we found that the R6BD does not bind to full-length Rad18 but does bind to some truncated forms of Rad18 (Figure 4). When we repeated this experiment with the W387A-R6BD, we found that the interaction is completely lost (Figure 4). This finding confirms that the R6BD can interact in trans with another region of the Rad6-Rad18 complex. This other region is either Rad6, an area near the N-termini of Rad18, or both.

**Figure 5:**
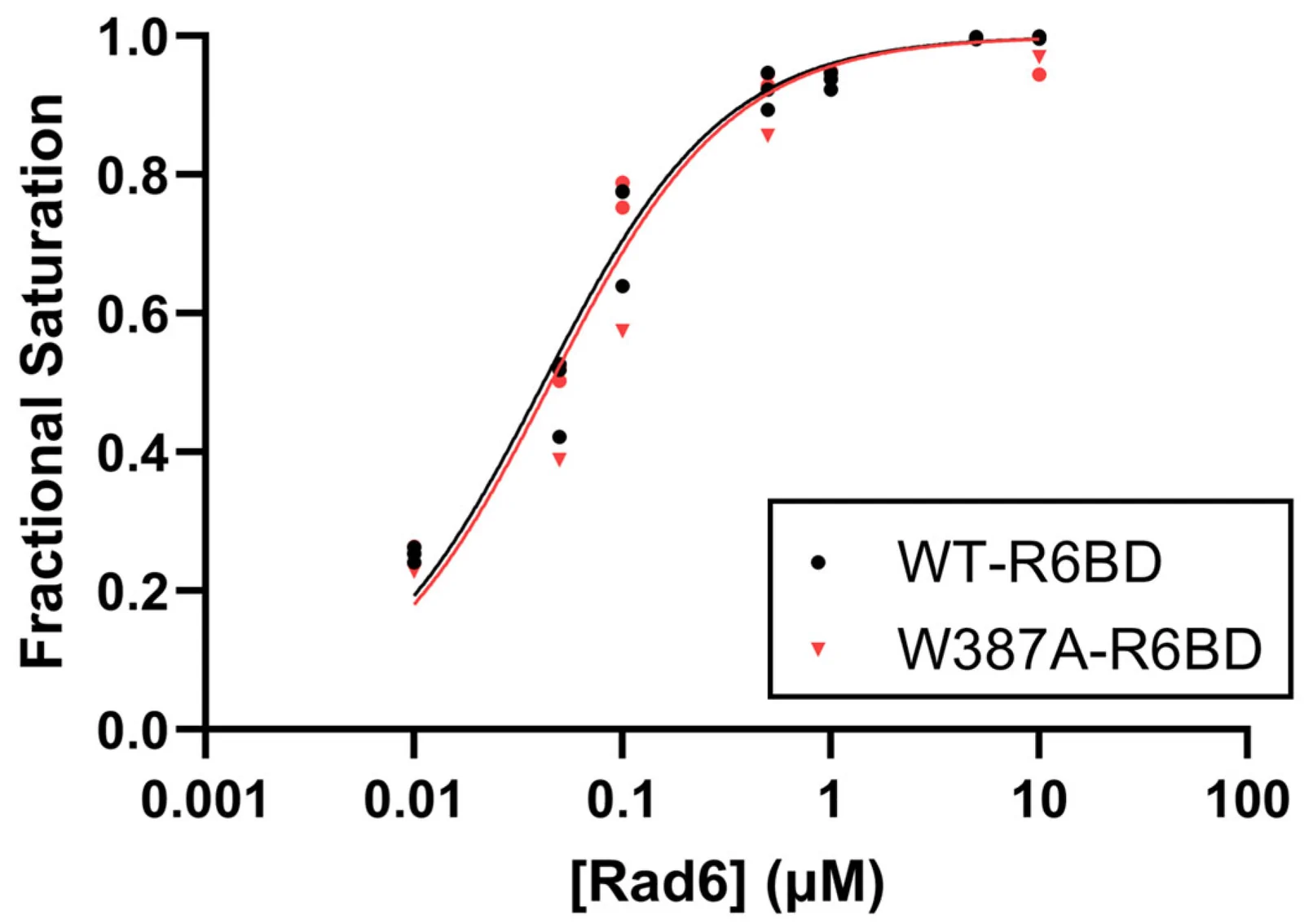
Isolated Rad6-Binding Domain (R6BD) Peptides Binding to Rad6. Normalized and processed biolayer interferometry (BLI) data demonstrating binding between isolated wild-type (black) or W387A mutant (red) R6BD peptides and Rad6 at various concentrations. One-site Adair equation fits yield K_d_^app^ equal to 42 ± 3 nM for wild-type and 45 ± 4 nM for the W387A mutant. Using a two-tailed, paired *t*-test, the two datasets were not significantly different (*p* = 0.309, n.s.).

To uncover which of these regions that the R6BD likely binds, we assessed the relative affinity between isolated peptides of the wild-type and W387A mutant R6BD to Rad6. Using biolayer interferometry (BLI), we found Rad6 interactions with affinities of 42 ± 3 nM and 45 ± 4 nM to the wild-type and W387A mutant, respectively (Figure 5). This finding rules out the possibility that within the Rad6-Rad18 complex the R6BD interacts only with Rad6. Instead, the R6BD likely interacts with both Rad6 and a region near the N-termini of Rad18. While the R6BD interacts strongly with Rad6, the W387A mutant does not affect the interaction to a detectable extent in the affinity. Thus, the effects of the W387A mutation are likely attributable to impaired interactions with the N-terminal region of Rad18, either alone or in the context of the Rad6–Rad18 complex.

### 3.4. Biological Function of the W387A Mutant Form of Rad18

**Figure 6:**
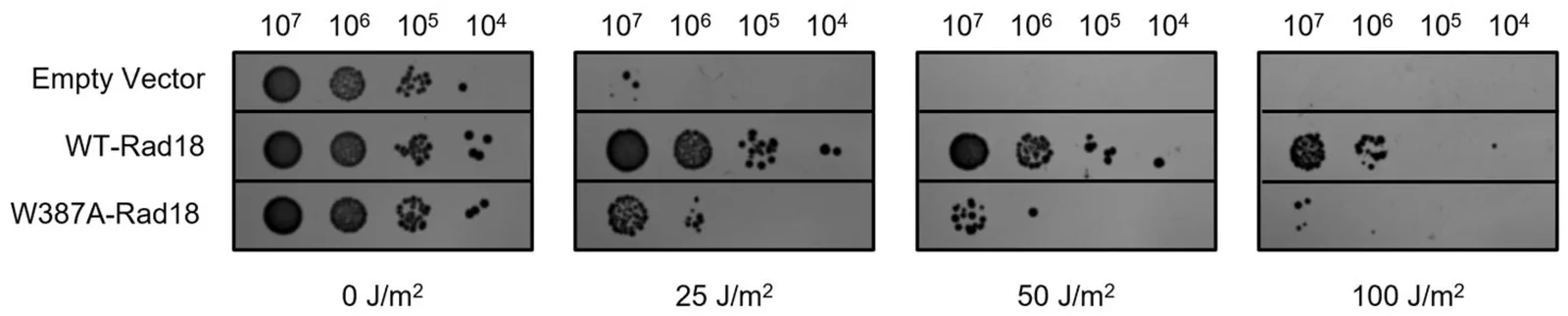
Genetic Complementation Analysis of Rad18 and its mutant (W387A). A representative stamping assay showing a serial dilution of yeast cells upon various dosages of UV radiation complemented with either an empty vector, wild-type Rad18, or Rad18 W387A.

Yeast strains that do not produce the Rad18 protein are extremely sensitive to ultraviolet (UV) radiation [33]. To test the impact of the W387A substitution in Rad18 on its ability to carry out DNA damage bypass in cells, we examined the UV sensitivity of yeast strains producing this mutant form of Rad18 (Figure 6). Semi-quantitative analyses were performed to assess the relative survival of cells (Supplemental Figure S1). Cells producing the W387A mutant form of Rad18 are more sensitive to UV radiation than cells producing wild-type Rad18. These cells, however, are not as sensitive to UV radiation as are cells producing no Rad18. This implies that while the mutant form of Rad18 supports a limited amount of DNA damage bypass in vivo, it does not support DNA damage bypass to the same extent as the wild-type protein.

**Figure 7:**
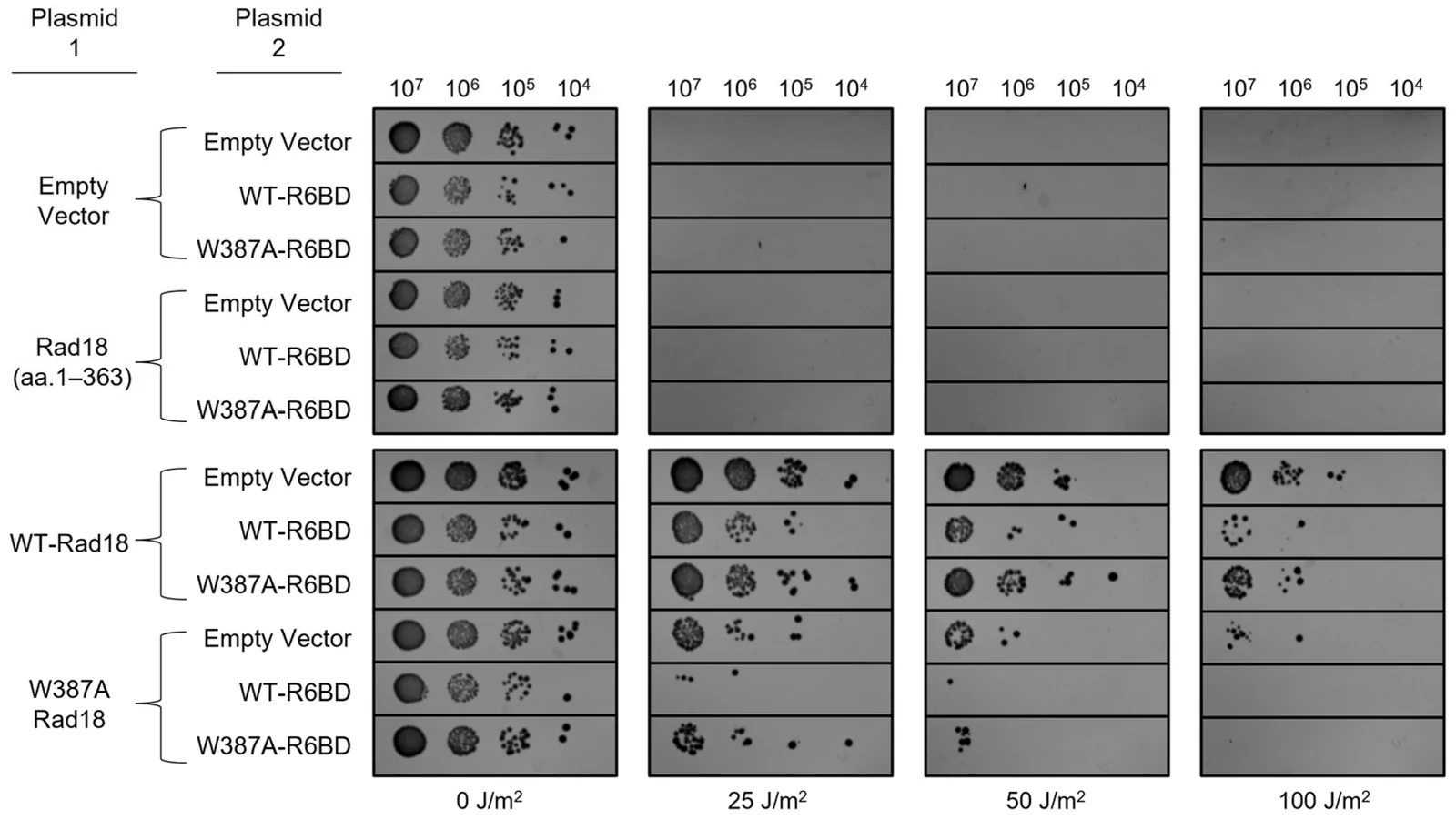
Genetic Complementation Analysis of Rad18 with the Rad6-Binding Domain (R6BD) in trans. A representative stamping assay showing a serial dilution of yeast cells upon various dosages of UV radiation of an empty vector, truncated mutant Rad18 (aa. 1–363), wild-type Rad18, or Rad18 W387A complemented with an empty vector, wild-type R6BD, or W387A R6BD.

To gain further insight into the biological effect of the W387A mutation, we evaluated UV sensitivity following in trans complementation of Rad18 (aa. 1–363) with either the wild-type or W387A Rad6-binding domain (R6BD) (Figure 7). Semi-quantitative analyses were performed to assess relative survival among the experimental groups (Supplemental Figures S2 and S3). As expected, expression of either the wild-type or W387A R6BD in the absence of Rad18 did not confer UV survival (Supplemental Figure S2). Surprisingly, expression of either R6BD variant also failed to increase UV survival in cells expressing truncated Rad18 (aa. 1–363) (Supplemental Figure S3). Together with our yeast two-hybrid results, these findings suggest that although the truncated Rad18 protein can interact with the wild-type R6BD in trans, the resulting complex is unable to support UV damage tolerance.

We next examined the effects of in trans complementation of full-length wild-type Rad18 or W387A Rad18 with either the wild-type or W387A R6BD (Figure 7). Semi-quantitative analyses were again performed to assess relative survival among experimental groups (Supplemental Figures S4 and S5). Unexpectedly, expression of the wild-type R6BD significantly reduced UV survival in cells expressing wild-type Rad18 compared with both the empty vector control and cells expressing the W387A R6BD (Supplemental Figure S4). A similar phenotype was observed in cells expressing W387A Rad18, where co-expression of the wild-type R6BD likewise decreased UV survival (Supplemental Figure S5). When considered alongside our yeast two-hybrid data, these results suggest that although the wild-type R6BD does not directly interact with full-length Rad18 when in trans, it can exert a dominant-negative effect on the pathway, potentially by sequestering Rad6 and limiting its availability for productive Rad18-mediated translesion synthesis.

## 4. Discussion

There are two non-mutually exclusive views about the role of the Rad6-Rad18 complex in recruiting TLS polymerases to stalled replication forks during DNA damage bypass [34,35,36,37,38,39]. In the first view, the Rad6-Rad18 complex plays an indirect role in recruiting TLS polymerases. In this view, the Rad6-Rad18 complex catalyzes the mono-ubiquitylation of PCNA, and the TLS polymerases subsequently bind the resulting ubiquitin-modified PCNA via their PCNA-interacting protein (PIP) motifs and their ubiquitin-binding motifs (UBMs)/UBZ motifs [34,35,36]. In the second view, the Rad6-Rad18 complex plays a direct role in recruiting TLS polymerases. In this view, the Rad6-Rad18 complex interacts with the TLS polymerases, and the TLS polymerases are recruited by riding “piggyback” on the Rad6-Rad18 complex as it is directed to stalled replication forks. This second view is supported by evidence showing that the Rad6-Rad18 complex constitutively binds pol η [37,38,39].

To better understand the potential roles of the Rad6-Rad18 complex in recruiting TLS polymerases, we have examined its oligomeric state and its interactions with TLS polymerases. Using mass photometry, we showed that the purified wild-type Rad6-Rad18 complex copurifies and forms various oligomeric states. Our yeast two-hybrid data have shown that the wild-type Rad18 binds pol η constitutively. By contrast, the wild-type Rad18 does not bind Rev1. Surprisingly, Rev1 (similar to Rfa1) binds to Rad18 when either a truncated form of Rad18 lacking the R6BD or a mutant form of Rad18 with a W387A substitution within the R6BD is used. This has led to the conclusion that the interactions between Rev1 or Rfa1 and the Rad6-Rad18 complex are regulated by auto-inhibition and that this auto-inhibition is released by the deletion of the R6BD or by specific amino acid substitutions within the R6BD of Rad18.

Auto-inhibition often involves an intramolecular interaction of the auto-inhibitory domain with another region of the protein necessary for its function [40,41,42,43]. If auto-inhibition occurs in regard to the interaction between Rad18 and Rev1, the R6BD would make an intramolecular interaction with another region of the Rad6-Rad18 complex that is necessary for interacting with Rev1, which sterically inhibits the ability of this region to interact with Rev1. Using yeast two-hybrid experiments, we tested this auto-inhibition model. We showed that while the isolated R6BD cannot bind to full-length Rad18, it can bind Rad18 proteins that lack its own R6BD. This result supports the view that this auto-inhibition involves an intramolecular interaction between the R6BD of Rad18 and another region of the Rad6-Rad18 complex, likely the Rad6 subunit, a region near the N-terminus of Rad18, or both.

**Figure 8:**
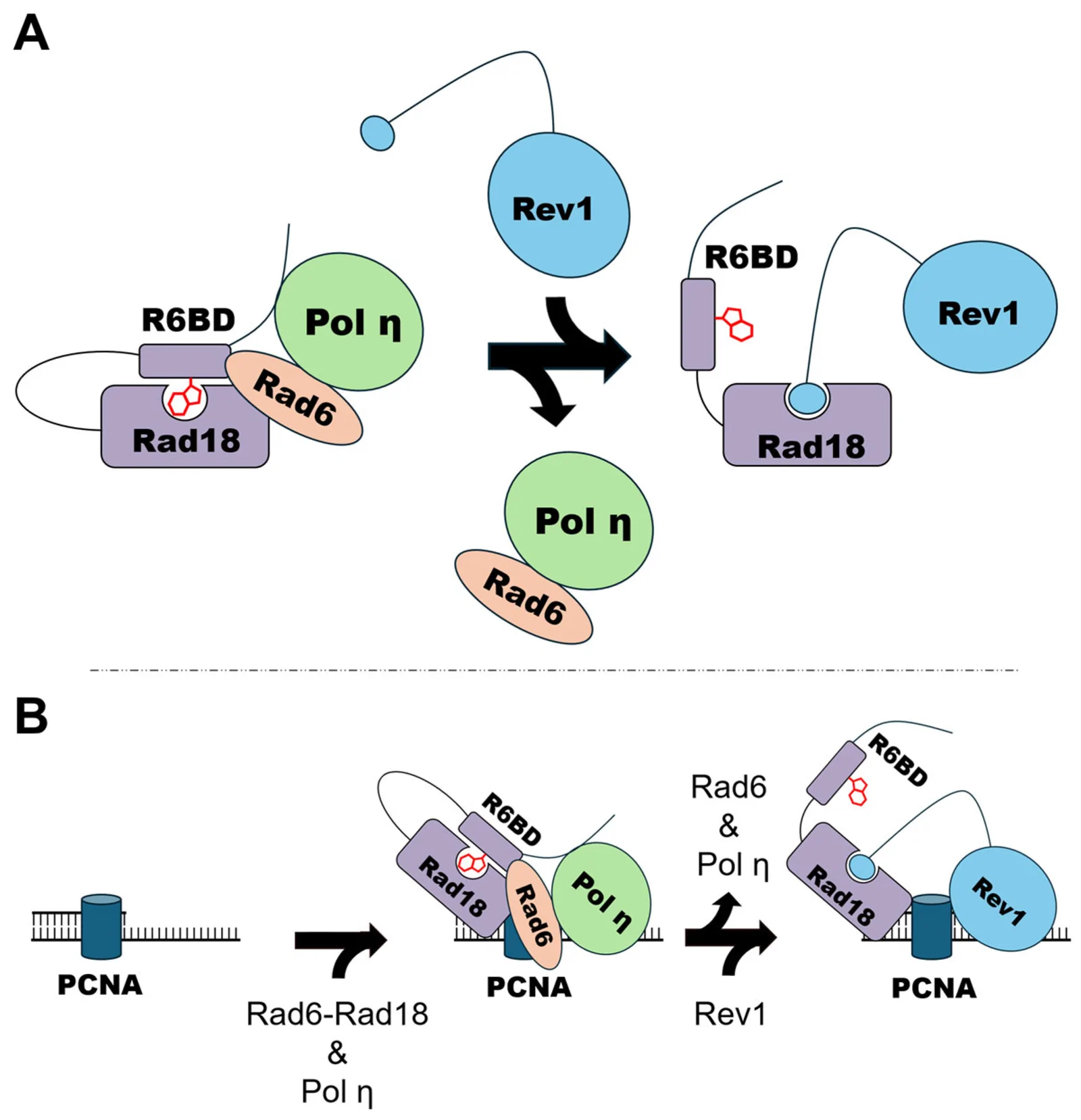
Sequential Recruitment Model of TLS Polymerases. Model describing the recruitment of pol η prior to Rev1. Rev1 is then recruited sequentially after pol η and Rad6 dissociate. This proposed model describes how the oligomeric state of the Rad6-Rad18 complex regulates sequential recruitment of TLS polymerases. (**A**) The top illustrates the oligomeric dynamics within the Rad6-Rad18 complex allowing Rev1 engagement. (**B**) The bottom depicts the sequential recruitment model within the context of TLS at a replication site.

To examine these possibilities, we first assessed the ability of Rad6 to co-purify with wild-type or W387A mutant Rad18. We found the wild-type Rad18 co-purifies and forms a series of punitive higher-order oligomeric states when in the presence of Rad6. In contrast, we found the W387A mutant Rad18 fails to co-purify with Rad6 and forms predominantly free monomers and homodimers. In addition, we also found that the wild-type and W387A mutant R6BD bind to Rad6 with the same affinity. From these findings, we have developed the following model (Figure 8A). Within the Rad6-Rad18 complex, the R6BD interacts with both a region near the N-termini of Rad18 and Rad6. This structural state is competent for binding pol η. It is also auto-inhibited with respect to Rev1 binding. When these intra-molecular interactions are disrupted, the protein changes conformations leading to pol η dissociation and Rev1 binding to a site on Rad18 that was previously occluded.

Interestingly, we have shown that the release of auto-inhibition is also accompanied by a change in the oligomeric state of the Rad6-Rad18 complex. The wild-type Rad6-Rad18 complex, which is auto-inhibited with respect to Rev1 and Rfa1 binding, comprises various oligomeric states likely in equilibrium with each other. Predominantly, we observed Rad18 monomers and one-to-one complexes of Rad6-Rad18. By contrast, the mutant W387A form of the Rad6-Rad18 complex dissociates into free Rad18 monomers and dimers, with no discernable species containing Rad6. We propose that the dissociation of the Rad6-Rad18 complex to free Rad18 monomers or Rad18 dimers accompanies the release of auto-inhibition. If this is the case, the Rad6-Rad18 complex would be only capable of binding Rev1 and Rfa1 with high affinity once this complex has dissociated to free Rad18 monomers or dimers. We note, however, that this model cannot distinguish whether auto-inhibition is relieved by the complete dissociation of Rad6 or a loss of interaction facilitated by the R6BD. Future studies will be necessary to distinguish between these possibilities.

Regardless of the mechanism by which auto-inhibition is released within the Rad6-Rad18 complex, the fact that there is auto-inhibition has important implications for TLS and for damage bypass. Pol η is best known for its ability to bypass thymine dimers and 8-oxoguanine lesions in a non-mutagenic (i.e., an error-free) manner [7,44,45]. Rev1, by contrast, is known for its ability to associate with pol ζ and bypass a wide range of DNA lesions in a mutagenic (i.e., an error-prone) manner [7,45,46]. Given that the Rad6-Rad18 complex binds pol η constitutively and binds Rev1 only after auto-inhibition is released, we propose a model by which Rad6-Rad18 reduces the likelihood of mutations by acting as a regulator to determine which TLS polymerase is recruited to stalled replication forks and when this recruitment occurs (Figure 8B). Because the Rad6-Rad18 complex binds pol η constitutively, it would preferentially recruit this non-mutagenic polymerase before auto-inhibition is released. This would make the non-mutagenic TLS polymerase pol η a “first responder” at stalled replication forks, where it can attempt to bypass the DNA lesion without competition from mutagenic TLS polymerases such as Rev1 and pol η. The mutagenic TLS polymerases would get a chance to attempt to bypass the lesion only after the release of auto-inhibition. This sequential recruitment model would reduce the likelihood of mutations during DNA damage bypass.

One important unanswered question is: what ultimately causes the dissociation of the Rad6-Rad18 complex and the concomitant release of auto-inhibition in the Rad6-Rad18 complex? There are a number of possibilities. First, the release of auto-inhibition may be triggered by binding to PCNA at the stalled fork. Second, auto-inhibition may be released by interactions of the Rad6-Rad18 complex with RPA or with DNA at the stalled fork. Third, the transfer of ubiquitin from the Rad6 E2 to the PCNA target may lead to the release of auto-inhibition. Further studies will be required to distinguish among the possible mechanisms of releasing auto-inhibition in the Rad6-Rad18 complex.

## 5. Conclusions

Recent studies have implicated Rad6-Rad18 as a key regulator of TLS polymerase recruitment to stalled replication forks. However, the mechanism by which Rad6-Rad18 recruits and coordinates TLS polymerases remains poorly understood. Here, we demonstrate that DNA polymerase η (pol η) binds constitutively to full-length Rad18, while the TLS polymerase Rev1 interacts only with truncated forms of Rad18. We also show that higher-order oligomerization of the Rad6-Rad18 complex auto-inhibits Rev1 binding. Together, our findings support a model in which the oligomeric state of the Rad6-Rad18 complex governs the sequential recruitment of TLS polymerases, with pol η recruited prior to Rev1. This mechanism provides a potential means of regulating polymerase exchange during TLS and may help minimize mutagenesis.

## Data Availability

The original contributions presented in this study are included in the article/Supplementary Material. Further inquiries can be directed to the corresponding author.

## Supplementary Materials

The supporting information can be downloaded at https://www.mdpi.com/article/10.3390/biom16081172/s1. Supplemental Figure S1. UV survival of yeast cells (Δrad18) producing wild-type or mutant W387A Rad18. Supplemental Figure S2. UV survival of yeast cells (Δrad18) producing wild-type or mutant R6BD. Supplemental Figure S3. UV survival of yeast cells (Δrad18) producing truncated Rad18 and wild-type or mutant R6BD in trans. Supplemental Figure S4. UV survival of yeast cells (Δrad18) producing wild-type Rad18 and wild-type or mutant R6BD in trans. Supplemental Figure S5. UV survival of yeast cells (Δrad18) producing W387A Rad18 and wild-type or mutant R6BD in trans. Supplemental Figure S6. Screening of the Rad6-binding Domain (R6BD) mutants. Supplemental Table S1: Plasmids for yeast two-hybrid assays. Original image of Polyacrylamide Gel can be found in Supplementary File.

## Abbreviations

The following abbreviations are used in this manuscript:

TLS	Translesion synthesis
pol	Polymerase
PCNA	Proliferating cell nuclear antigen
UBZ	Ubiquitin-binding, zinc-binding
R6BD	Rad6-binding domain
SAP	SAF-A/B, Acinus, and PIAS
SIM	SUMO-interacting motif
UV	Ultraviolet
RPA	Replication protein A
