# Supplementary material for: The Oligomeric State of Rad6-Rad18 and Its Interactions with Translesion Synthesis Proteins
Source: Biomolecules. 2026 Aug 12;16(8):1172. doi: 10.3390/biom16081172 (PMC13510358; doi:10.3390/biom16081172)
Supplement: Supplementary file 1 [file biomolecules-16-01172-s001.zip › biomolecules-4475684 Gel original image.pdf]

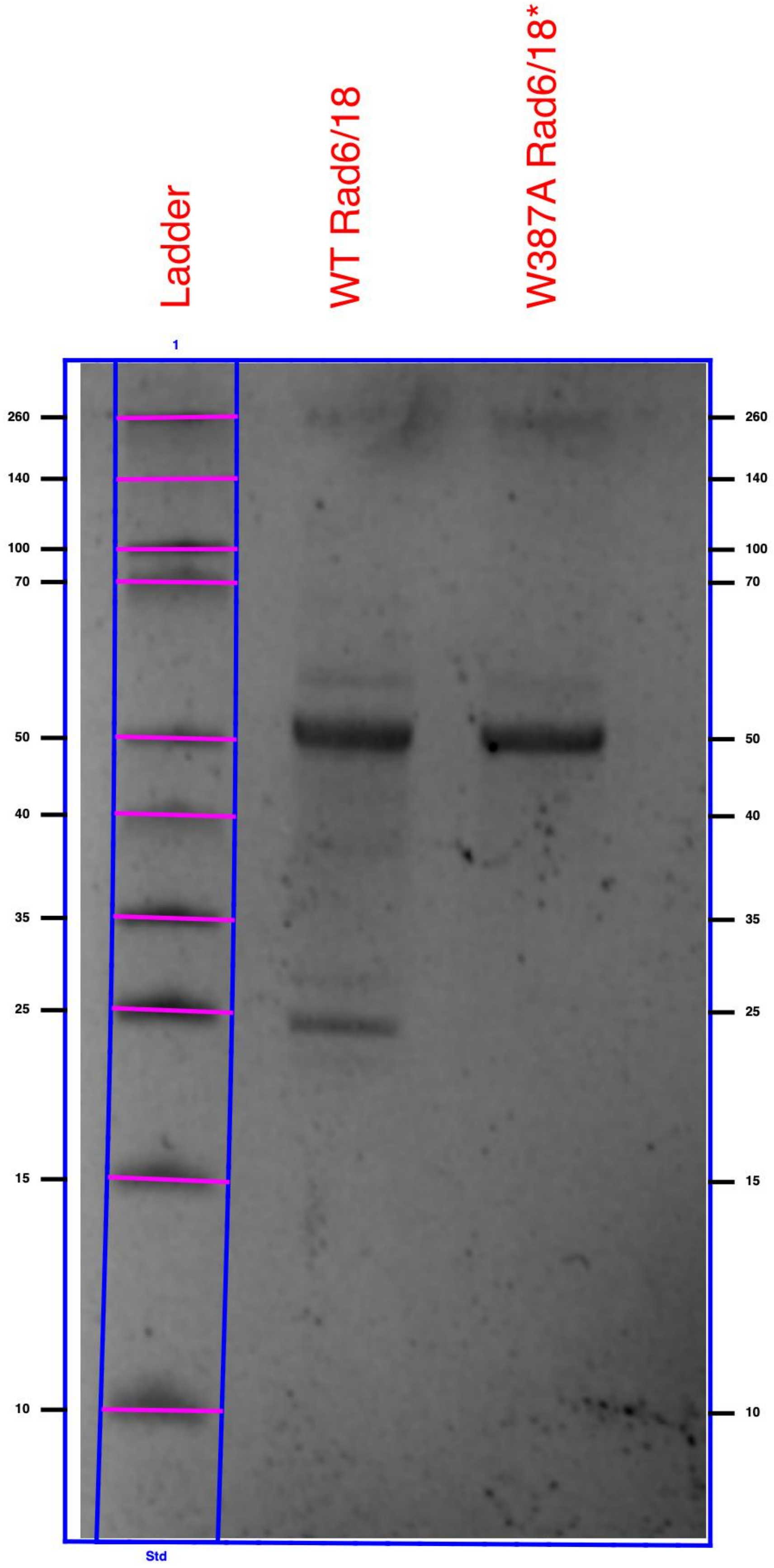
Ladder
WT Rad6/18
W387A Rad6/18*
1
260
140
100
70
50
40
35
25
15
10
Std
