# Supplementary material for: The Oligomeric State of Rad6-Rad18 and Its Interactions with Translesion Synthesis Proteins
Source: Biomolecules. 2026 Aug 12;16(8):1172. doi: 10.3390/biom16081172 (PMC13510358; doi:10.3390/biom16081172)
Supplement: Supplementary file 1 [file biomolecules-16-01172-s001.zip › biomolecules-4475684-supplementary.pdf]

Supplemental Table S1: Plasmids for yeast two-hybrid assays

Plasmid Name	Short Description	Long Description
pKW835	fl-Rad18	yRad18 in pGAD vector; yeast-two hybrid assays
pKW836	tRad18 (aa. 1–363)	yRad18 (aa. 1–363) in pGAD vector; yeast-two hybrid assays
pKW837	tRad18 (aa. 1–255)	yRad18 (aa. 1–255) in pGAD vector; yeast-two hybrid assays
pKW838	tRad18 (aa. 1–217)	yRad18 (aa. 1–217) in pGAD vector; yeast-two hybrid assays
pKW839	tRad18 (aa. 1–186)	yRad18 (aa. 1–186) in pGAD vector; yeast-two hybrid assays
pKW840	tRad18 (aa. 1–98)	yRad18 (aa. 1–98) in pGAD vector; yeast-two hybrid assays
pKW841	W387A-Rad18	W387A-Rad18 in pGAD vector; yeast-two hybrid assays
pKW842	yRad30	yRad30 in pGBKT7 vector; yeast-two hybrid assays
pKW843	yRev1	yRev1 in pGBKT7 vector; yeast-two hybrid assays
pKW844	Rfa1	Rfa1 in pGBKT7 vector; yeast-two hybrid assays
pKW845	R6BD	Wildtype R6BD (aa. 365–425) in pGBKT7 vector; yeast-two hybrid assays
pKW846	tRad18 (aa. 1–317)	yRad18 (aa. 1–317) in pGAD vector; yeast-two hybrid assays
pKW848	W387A-R6BD	W387A-R6BD (aa. 365–425) in pGBKT7 vector; yeast-two hybrid assays

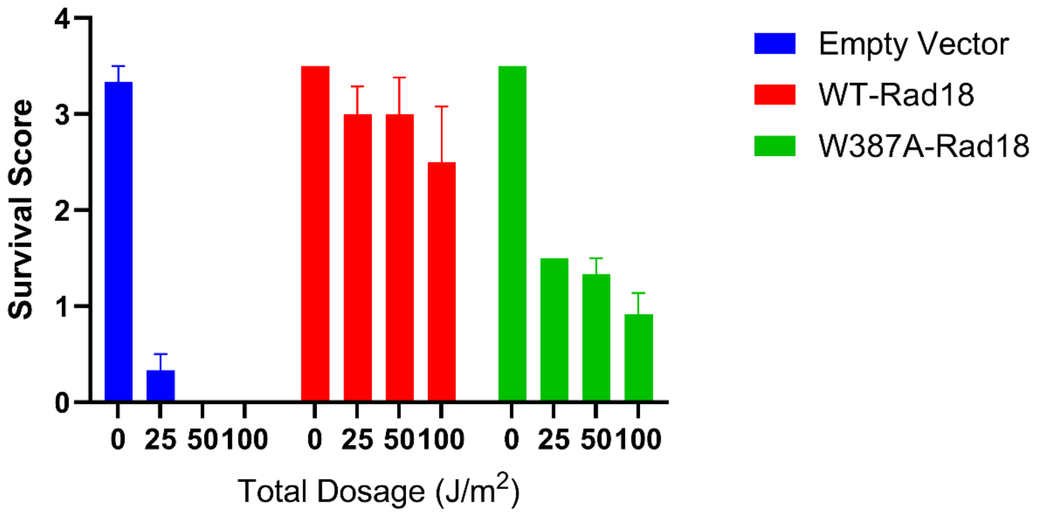

Supplemental Figure S1. UV survival of yeast cells (Δ*rad18*) producing wild-type or mutant W387A Rad18. Semi-quantitative analysis of the yeast stamping assay showing survival of BY4742 Δ*rad18* cells following exposure to increasing doses of UV radiation. Cells were transformed with plasmids expressing wild-type Rad18, the Rad18 W387A mutant, or an empty vector for control. Survival was scored as described in the materials and methods.

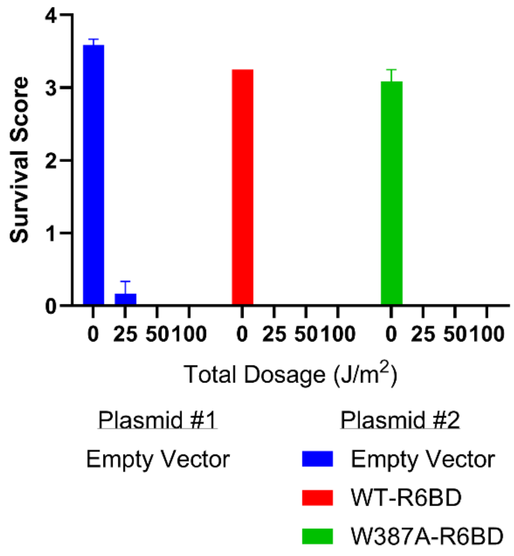

Supplemental Figure S2. UV survival of yeast cells (Δ*rad18*) producing wild-type or mutant R6BD. Semi-quantitative analysis of the yeast stamping assay showing survival of BY4742 Δ*rad18* cells following exposure to increasing doses of UV radiation. Cells were transformed with an empty vector and a second plasmid expressing either wild-type R6BD or the R6BD W387A mutant, or with a second empty vector as a control. Survival was scored as described in the materials and methods.

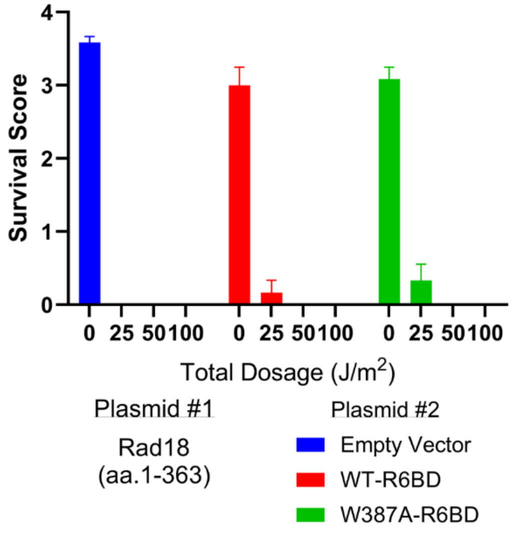

Supplemental Figure S3. UV survival of yeast cells (Δ*rad18*) producing truncated Rad18 and wild-type or mutant R6BD *in trans*. Semi-quantitative analysis of the yeast stamping assay showing survival of BY4742 Δ*rad18* cells following exposure to increasing doses of UV radiation. Cells were transformed with a plasmid expressing truncated Rad18 (residues 1–363) and a second plasmid expressing either wild-type R6BD or the R6BD W387A mutant, or with an empty vector as a control. Survival was scored as described in the materials and methods.

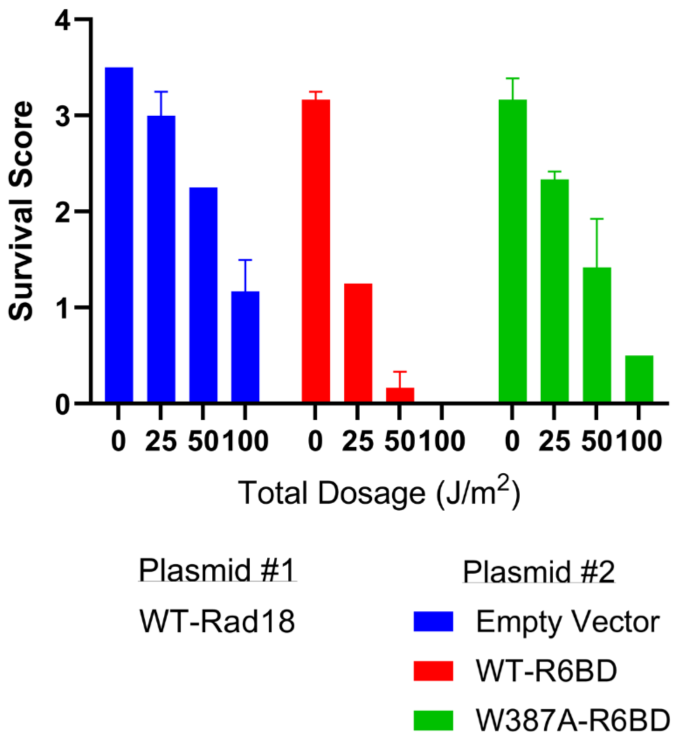

Supplemental Figure S4. UV survival of yeast cells (Δ*rad18*) producing wild-type Rad18 and wild-type or mutant R6BD *in trans*. Semi-quantitative analysis of the yeast stamping assay showing survival of BY4742 Δ*rad18* cells following exposure to increasing doses of UV radiation. Cells were transformed with a plasmid expressing wild-type Rad18 and a second plasmid expressing either wild-type R6BD or the R6BD W387A mutant, or with an empty vector as a control. Survival was scored as described in the materials and methods.

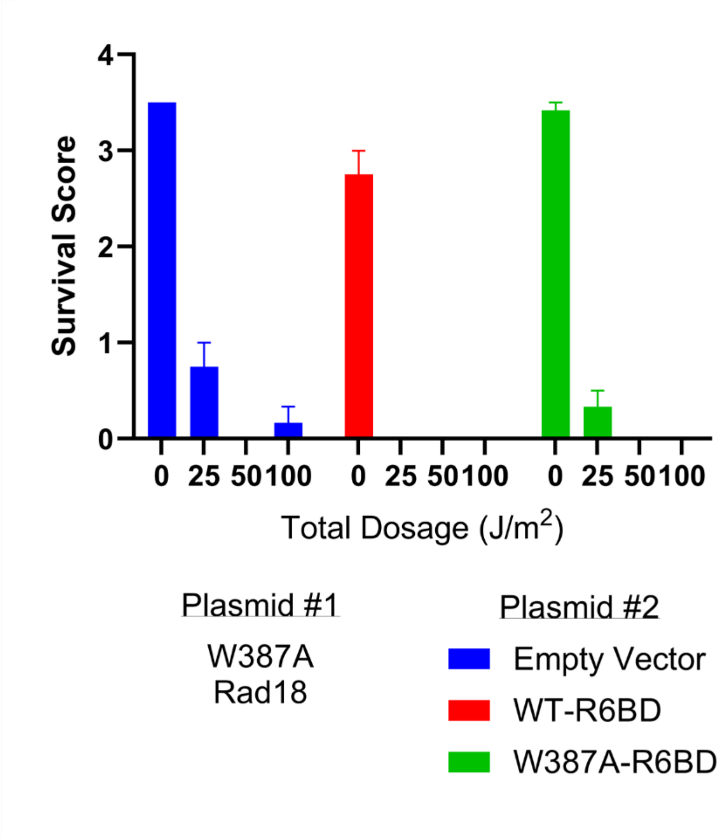

Supplemental Figure S5. UV survival of yeast cells (Δ*rad18*) producing W387A Rad18 and wild-type or mutant R6BD *in trans*. Semi-quantitative analysis of the yeast stamping assay showing survival of BY4742 Δ*rad18* cells following exposure to increasing doses of UV radiation. Cells were transformed with a plasmid expressing Rad18 W387A mutant and a second plasmid expressing either wild-type R6BD or the R6BD W387A mutant, or with an empty vector as a control. Survival was scored as described in the materials and methods.

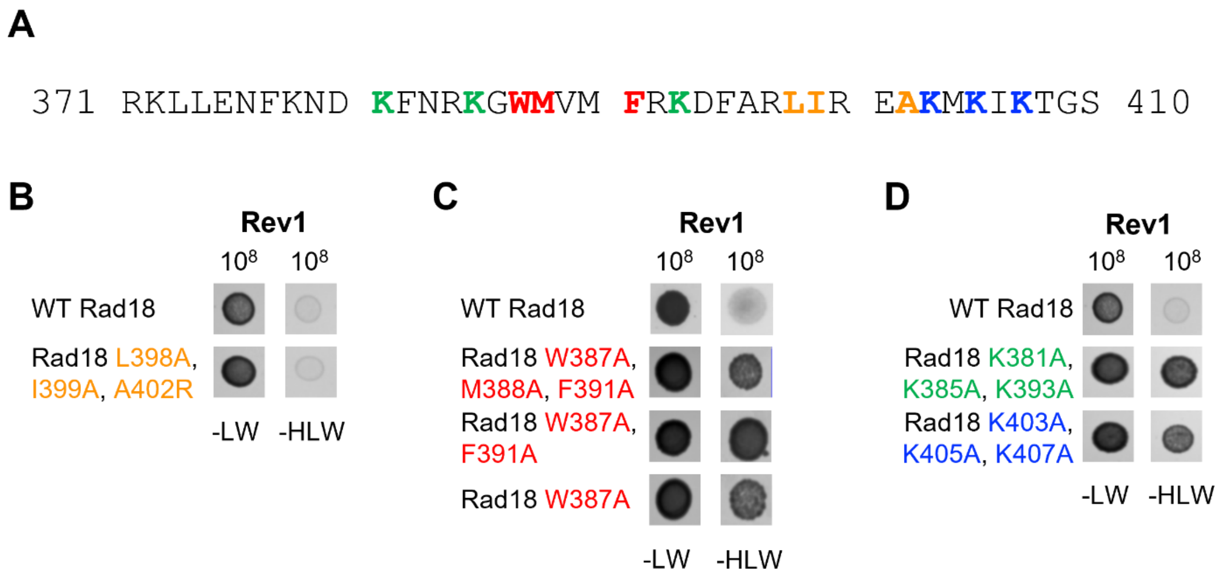

Supplemental Figure S6. Screening of the Rad6-binding Domain (R6BD) mutants (**A**) Yeast Rad18 sequence of the Rad6-binding domain (residues 371-410) with colors representing the sites corresponding to panels B-D. (**B**) Yeast two-hybrid assay results for the Rad18 L398A/I399A/A402R triple mutant. (**C**) Yeast two-hybrid assay results for the Rad18 W387A/M388A/F391A triple mutant, the Rad18 W387A/F391A double mutant, and the Rad18 W387A single mutant. (**D**) Yeast two-hybrid assay results for the Rad18 K381A/K385A/K393A triple mutant and the Rad18 K402A/K405A/K407A triple mutant.
